# Selenium Nanoparticles Modulate Steroidogenesis-Related Genes and Improve Ovarian Functions via Regulating Androgen Receptors Expression in Polycystic Ovary Syndrome Rat Model

**DOI:** 10.1007/s12011-023-03616-0

**Published:** 2023-03-16

**Authors:** Ahmed B. E. Abdallah, Mohammed A. El-Ghannam, Azza A. Hasan, Lamiaa G. Mohammad, Noura M. Mesalam, Radwa M. Alsayed

**Affiliations:** 1 Department of Physiology, Faculty of Medicine, Zagazig University, Zagazig, Egypt; 2 Department of Pharmaceutics, Faculty of Pharmacy, Zagazig University, Zagazig, Egypt; 3 Biological Applications Department, Nuclear Research Center, Egyptian Atomic Energy Authority, 13759 Cairo, Egypt

**Keywords:** PCOS, Selenium nanoparticles, Steroidogenesis, mRNA expression, Androgen receptors, Ovarian histopathology and immunohistochemistry

## Abstract

Polycystic ovary syndrome (PCOS) occurs during the reproductive period in women and is characterized by reproductive, endocrine, and metabolic disorders. Androgen plays a decisive role in its pathogenesis due to the interaction between hyperandrogenism and insulin resistance, which might be improved by selenium nanoparticles (SeNPs). The present study aimed to clarify the effect of SeNPs on androgen synthesis and action in the PCOS model and the resulting effect on ovarian function. Fifty-five 7-week-old female albino rats (90–105 g) were divided equally into five groups: control (C), fed a standard diet for 11 weeks; high-fat diet (HFD) group, fed HFD for 11 weeks; HFD and letrozole (L) (HFD + L), fed HFD for 11 weeks and administrated orally with L, at a daily dose of 1 mg/kg BW, for three weeks from the 7th to 9th week of the trial; HFD + L + 0.1SeNPs and HFD + L + 0.2SeNPs groups, treated the same as HFD + L group and orally gavaged SeNPs at daily doses of 0.1 and 0.2 mg/kg BW, respectively, during the last 14 day of the experiment. Daily determination of estrous cycle was performed, and at the end of the experimental period, BMI, serum glucose, insulin, HOMA-IR, lipid profile, sex hormones, TNF-α, IL6, oxidative stress biomarkers, ovarian mRNA expression of different proteins and enzymes involved in steroidogenesis, pathological examination, and immunohistochemical staining for androgen receptor (AR) were evaluated. Treatment of SeNPs restored estrous cyclicity, decreased BMI, and insulin resistance, improved dyslipidemia, reduced serum testosterone, and improved ovarian histopathology in PCOS rats. Furthermore, the anti-inflammatory and antioxidant impacts of SeNPs were remarkably noticed. Administration of SeNPs decreased androgen synthesis and expression of ovarian AR protein by decreasing the mRNA expression of STAR, Cyp11A1, Cyp17A1, and HSD17B3 and increasing the expression of Cyp19α1*.* Conclusively, SeNPs decreased androgen synthesis and blocked the vicious circle initiated by excessive androgen secretion via decreased AR expression. Thus, it may effectively treat PCOS cases by eliminating its reproductive, endocrine, and metabolic dysfunctions.

## Introduction

Polycystic ovary syndrome (PCOS) is a complex endocrine, reproductive, and metabolic disorder that afflicts ~ 6–20% of females of reproductive age, characterized by hyperandrogenemia, irregular menstrual cycles, polycystic ovaries, lack of ovulation, amenorrhea, and infertility [1]. PCOS-associated hyperandrogenism leads to the early luteinization of granulosa cells (GCs), which affects folliculogenesis (abnormal follicular development), causing follicle atresia and hence ovulation [2]. Numerous other metabolic and clinical symptoms of PCOS have been reported, such as insulin resistance and diabetes, cardiovascular disease, hypertension, oxidative stress, endometrial hyperplasia, and breast cancer [2, 3].

Lazaros et al. [4] revealed that estradiol is generated in the ovary through the conversion of C19 androgens derived from theca cells under the influence of aromatase produced by granulosa cells. Caldwell et al. [5] revealed that aromatase catalyzes the rate-determining step during the biosynthesis of estrogens from androgens, and disturbances in the secretion and metabolism of estrogens and androgens could be linked to decreased aromatase activity resulting in polycystic ovaries. In PCOS, the deficiency in aromatase enzyme activity is thought to disturb intraovarian steroidogenesis and induce ovarian failure. Furthermore, PCOS is triggered either by releasing excessive luteinizing hormone (LH) or high insulin levels or their combination [6]. PCOS women have an increased LH/FSH ratio which could be due to the increased frequency of hypothalamic gonadotrophin-releasing hormone (GnRH) pulses, which enhances androgen production and decreases maturation of the ovarian follicles via overstimulation of ovarian theca cells [7].

Although the etiology of PCOS is still uncertain, the pathogenesis and prevalence of PCOS are controlled by various genetic and environmental factors. Approximately 75% of PCOS women have high androgen levels, which may be genetically acquired. The androgen synthesis pathway involves several important proteins, including the steroidogenic acute regulatory protein (StAR) and the Cytochrome P450 side chain cleavage enzyme (CYP11A1). The CYP11 gene, located on chromosome 15q24, codes for the enzyme CYP11A1 and is crucial for steroid synthesis [8]. Miller [9] has shown that deletion of the CYP11 gene in rabbits abolishes steroid synthesis, suggesting that steroidogenesis begins with the action of this enzyme. There is also an association between the CYP11 gene and polycystic ovary syndrome. A study by Gharani et al. [10] found that a specific pentanucleotide in the 5′-untranslated region (UTR) of the CYP11 gene is associated with PCOS and elevated serum testosterone levels in women with PCOS. Another important enzyme in the androgen synthesis pathway is the cytochrome P450 17α-hydroxylase-17, 20-lyase, which is coded by the CYP17 gene located on chromosome 10q24-q25. This enzyme has both hydroxylase and lyase activity and converts pregnenolone to dehydroepiandrosterone [11]. Alterations in the P450 CYP17 enzyme are thought to be one of the causes of ovarian hyperandrogenism found in PCOS [12]. Furthermore, the enzyme P450 aromatase, encoded by the CYP19 gene located at 15p21, is present in a complex state consisting of the cytochrome P450 aromatase (P450arom), nicotinamide adenine dinucleotide phosphate (NADPH), and cytochrome P450 reductase. This enzyme catalyzes the conversion of androgens into estrogens. Erickso et al. [13] have shown that aromatase expression is decreased in granulosa cells obtained from women with PCOS.

In addition, unhealthy lifestyles, diet, physical activity, and insulin resistance have crucial roles in its pathogenesis [14]. Recently, it has been demonstrated that letrozole and high-fat diet (HFD) PCOS rat model showed a phenotype very similar to that of human PCOS, providing a highly suitable model for the study of PCOS [15]. Although POCS can be managed through several chemical or hormonal therapies, these remedies are accompanied by severe and various side effects such as psychological disturbances, arthritis, lactic acidosis, irregular uterine bleeding, cardiovascular and thromboembolic risks, renal and hepatic toxicities, hyperplasia, uncertain risks, in addition to their considerable costs [16].

Nanotechnology, a revolutionary tool with cutting-edge breakthroughs, could be one of the most promising solutions. Nanoparticles have unique physical and chemical properties; their nano-sizes make them acquire several biological activities and could be used in disease treatment [17–19]. Interestingly, nanoscale elemental selenium attracted attention due to its biological activities such as antioxidant, scavenging of reactive oxygen species through enhancing the activity of GPx and selenoproteins, anti-inflammatory, and antihyperglycemic [20, 21]. These effects might be attributed to the small size and large surface area of nanoparticles, acquiring the high absorption efficiency. In addition, selenium nanoparticles (SeNPs) have high bioavailability and low toxicity sevenfold than sodium selenite [22].

Recent studies have demonstrated that Se and SeNPs improve insulin resistance in PCOS women and rat models [23, 24]. Nevertheless, the effect of SeNPs on androgen production in PCO models has not yet been investigated. Therefore, the present study aimed to shed more light on the cellular mechanism of SeNPs in regulating androgen production and their mode of action through regulating steroidogenesis enzymes and androgen receptors protein expression and the resulting effect on ovarian function in the PCOS rat model.

## Materials and Methods

### Chemicals and Drugs

Letrozole (L) was obtained from ACDIMA International Co., Egypt. Aqueous carboxy methyl cellulose (CMC), ascorbic acid, chitosan, and selenium dioxide were obtained from Sigma Chemical, St. Louis, MO, Sigma-Aldrich, USA.

### Preparation of SeNPs

SeNPs were chemically synthesized, as described by Zhang et al. [25]. In brief, SeNPs were prepared by reducing selenium dioxide solution with ascorbic acid in the presence of chitosan. The obtained SeNPs were characterized by transmission electron microscopy (TEM).

### Characterization of SeNPs

SeNPs were analyzed by TEM by placing a drop of the nanoparticle suspension on carbon-coated copper grids. Under an infrared lamp, the samples were dried, and then the images were recorded using TEM Philips CM 200 instrument with an operating voltage of 80 kV and resolution of up to 2.4 A.

### Animals and Ethics Statement

The experimental protocol was approved by the Institutional animal care and use committee of Zagazig University (ZU-IACUC/3/F/111/2019) and Egyptian Atomic Energy Authority (2PA/23). Fifty-five 7-week-old healthy female albino rats weighing 90–105 g were purchased from the animal house of the Faculty of Veterinary Medicine, Zagazig University. The experiment was conducted in the animal house of the Faculty of Medicine, Zagazig University, under hygienic and standard environmental conditions (24 ± 2 °C, 12-h light/dark cycle). The animals were kept in steel wire cages (40 × 28 × 18 cm) with free access to standard food pellets and water.

### Experimental Scheme and Induction of PCOS

Animals were adapted to laboratory conditions for 1 week before the beginning of the experiment and then allocated into five groups (11 rats/group). The first group served as a control (C), administered with 0.5% CMC and fed a standard diet (25.8% protein, 62.8% carbohydrate, and 11.4% fat) for 11 weeks, according to [26]. The standard diet was supplemented with a selenium free mineral-vitamin premix and offered *ad libitum* to each group. The second group (HFD) was administered with 0.5% CMC and fed HFD for 11 weeks, consisting of 20% protein, 35% carbohydrates, and 45% fat, mainly as saturated fat [27]. The third group (HFD + L) was fed HFD for 11 weeks and administrated orally with letrozole, at a daily dose of 1 mg/kg BW dissolved in 0.5% CMC, for 3 weeks from the 7th to 9th week of the trial [28]. The fourth (HFD + L + 0.1SeNPs) and fifth groups (HFD + L + 0.2SeNPs) were treated the same as the third group and orally gavaged SeNPs, at daily doses of 0.1 and 0.2 mg/kg BW, respectively, during the last 14 day of the experiment [29].

### Determination of Estrous Cycle

Vaginal smears were performed daily at 10 am to determine estrous phases [30]. The vagina was flushed with saline, the fresh unstained vaginal fluids were microscopically examined during the experimental period, and the mean frequency of diestrus, metestrus, proestrus, and estrus was compared between the groups. Cycles with 4–5 days were considered regular, while persistent estrus, the presence of prolonged cornified cells in the smears for at least two consecutive estrous cycles, indicated the development of follicular cysts and successful induction of PCOS [28].

### Determination of Body Mass Index (BMI)

Animals were weighed on the first and last days of the experiment. The initial and final BMI was calculated following this equation: body weight (gm)/length^2^ (cm^2^).

### Blood and Tissue Sampling

At the end of the experiment, animals fasted overnight then blood samples were collected from the orbital sinus under ether anesthesia. Blood samples were centrifuged at 1500 × g for 15 min then sera were separated and stored at − 20 °C until the biochemical analysis. Subsequently, the animals were sacrificed, and ovaries were excised, defatted, cleaned in saline, and weighed. One part of the ovary was weighed and homogenized in phosphate buffer saline, centrifuged at 10,000 × g for 10 min, then the clear supernatant was separated and stored at − 80 °C for analysis. The second part of the ovaries was immediately fixed in 10% neutral buffered formalin for histopathological and immunohistochemical evaluation.

### Biochemical Assays

#### Quantification of Metabolites

Serum fasting blood glucose and insulin levels were measured using (GOD-PAP)-Liquizyme kit (Biotechnology, Egypt) and ELISA kit (Sigma-Aldrich Chemie GmbH, USA) following the methods described by Tietz [31] and Temple et al. [32], respectively. The detection of homeostasis model assessment of insulin resistance (HOMA/IR) was conducted using the following equation [HOMA-IR = insulin (µU/ml) × glucose (mg/dl)/405] [33].

### Lipid Profile Assays

Serum triglycerides (TG), total cholesterol (TC), and high-density lipoprotein (HDL) levels were determined colorimetrically (Spectronic 1201, Milton Roy, Ivyland, PA, USA) following the manufacturer’s instructions (Spinreact Co., Girona, Spain)*.*

### Hormonal Assay

The serum levels of testosterone, estradiol (E2), progesterone, luteinizing hormone (LH), and follicle-stimulating hormone (FSH) were measured by radioimmunoassay (Beijing North Institute of Biotechnology Co., Ltd., Beijing, China) according to the manufacturer’s instructions.

### Oxidative Stress and Inflammatory Cytokine Assays

The ovarian levels of glutathione peroxidase (GPx) and malondialdehyde (MDA), a measure of lipid peroxidation, were assessed using rat kits (Catalog Number Sigma-MAK085, Sigma-Aldrich, USA) [34].

The serum levels of inflammatory cytokines (TNF-α and IL-6) were measured using immunoassay kits (BioSource International Inc., CA, USA) [35, 36].

### Real-time Quantitative PCR (qRT-PCR) Analysis

Total RNA was isolated from frozen ovarian tissues using TRIzol RNA Isolation Reagents. Total RNA was quantified using a NanoDrop® ND–1000 Spectrophotometer (NanoDrop Technologies; Wilmington, DE, USA) for 1.5 µl of the RNA. Total RNA (2 μg) was reverse transcribed using the High-Capacity cDNA Reverse Transcription Kit (Applied Biosystems™, USA). Furthermore, real-time PCR was performed in an Mx3005P Real-Time PCR System (Agilent Stratagene, USA) using TOPreal™ qPCR 2X PreMIX (SYBR Green with low ROX) (Cat. # P725 or P750) (Enzynomics, Korea) following the manufacturer’s instructions. Primers were procured from Sangon (Sangon Biotech, Shanghai, China), and sequences are shown in Table 1. The relative quantity of each gene in the ovarian tissues of rats was normalized to glyceraldehyde-3-phosphate dehydrogenase (GAPDH), and the relative expression fold change was calculated by calculating ΔΔCt [37].

**Table 1:** Primer sequences and target genes for SYBR green RT-PCR

Gene	Forward	Reverse	Accession No
GAPDH	GCATCTTCTTGTGCAGTGCC	GGTAACCAGGCGTCCGATAC	NM_017008.4
STAR	CCCAAATGTCAAGGAAATCA	AGGCATCTCCCCAAAGTG	NM_031558.3
CYP11A1	AAGTATCCGTGATGTGGG	TCATACAGTGTCGCCTTTTCT	NM_017286.3
CYP17A1	TGGCTTTCCTGGTGCACAATC	TGAAAGTTGGTGTTCGGCTGAAG	NM_012753.2
HSD17B3	AGTGTGTGAGGTTCTCCCGGTACCT	TACAACATTGAGTCCATGTCTGGCCAG	NM_054007.1
CYP19A1	GCTGAGAGACGTGGAGACCTG	CTCTGTCACCAACAACAGTGTGG	NM_017085.2

*STAR*, Steroidogenic acute regulatory protein; *CYP11A1*, Cytochrome P450 Family 11 Subfamily A Member 1; *CYP17A1*, Cytochrome P450 Family 17 Subfamily A Member 1; *CYP19A1*, Cytochrome P450 Family 19 Subfamily A Member 1; *HSD17B3*, 17-beta hydroxysteroid dehydrogenase 3; *GAPDH*, Glyceraldehyde 3-phosphate dehydrogenase

### Histopathological Examination of Ovarian Tissues

Ovarian tissues were kept in 10% neutral buffered formalin for fixation. Five *μ* thickness slices were cut from paraffin blocks using a microtome. A routine tissue processing protocol was followed to obtain hematoxylin and eosin (H&E) stained tissue slides [38].

### Immunohistochemical Investigation of Androgen Receptors

Immunohistochemistry (IHC) was done using rabbit polyclonal primary antibody against androgen receptor (AR) (Abcam, UK; dilution 1:500; Cat No ab3510). For IHC quantification, representative ovarian sections from 11 rats in each group were taken using a bright field microscope. The photomicrographs of AR staining were analyzed using image analysis software and expressed as AR stain area.

### Statistical Analysis

The results of the present study were subjected to ANOVA procedures appropriate for a completely randomized design using the GLM procedures of the SPSS. Shapiro–Wilk and Levene tests were used to test the normal distribution of data as well as the homogeneity of variance. Tukey’s multiple comparison test was used to determine the significant differences between groups.

## Results

### SeNPs Characterization

**Fig. 1:**
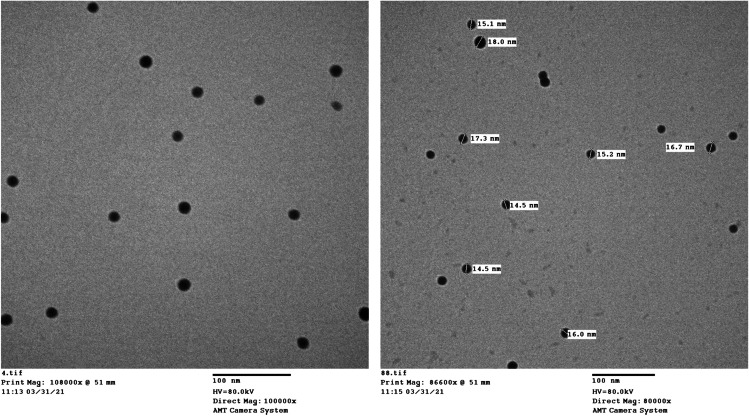
Representative transmission electron microscopy analysis of prepared selenium nanoparticles (SeNPs) showing their size and shape

The TEM of SeNPs observed that its mean diameter ranged between 15 and 25 nm, indicating the efficient synthesis of SeNPs with a spherical structure (Fig. 1).

### Body Mass Index

**Fig. 2:**
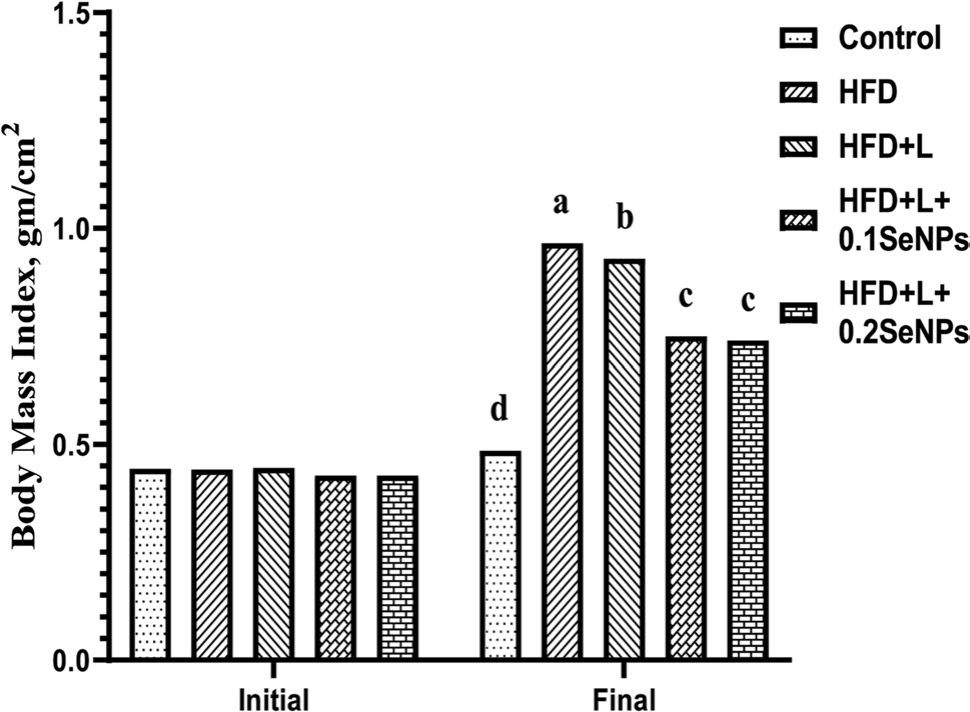
Initial and final body mass index of female albino rats treated with selenium nanoparticles (SeNPs) in a polycystic ovary syndrome model. HFD, high-fat diet; L, letrozole

As illustrated in Fig. 2, initial BMI did not differ among experimental groups at the beginning of the experiment. Final BMI increased (*P* <0.05) in all treated groups compared to the control. The highest value of final BMI was recorded in HFD, followed by HFD + L.

### Serum Metabolites and Lipid Profile

The induction of PCOS in HFD and HFD + L groups elevated (*P*  ﻿< 0.01) serum levels of TC, TG, and LDL and reduced HDL concentration compared to the normal group (Table 2). A significant increase (*P* < 0.01) in the serum levels glucose, insulin, and HOMA_IR was noticed when compared to normal control group. Values of all the aforementioned parameters were improved (*P* ﻿< 0.01) by the treatment with both levels of SeNPs.

**Table 2:** Effect of selenium nanoparticles (SeNPs) on serum glucose, insulin, and lipid profile and HOMA_IR in polycystic ovary syndrome rat model

Items	Glucose (mg dl^−1^)	Insulin (µIU ml^−1^)	HOMA_IR	TC (mg dl^−1^)	TG (mg dl^−1^)	LDL (mg dl^−1^)	HDL (mg dl^−1^)
C	80.90^c^	18.02^c^	3.552^c^	78.56^c^	71.97^c^	26.31^c^	37.86^a^
HFD	187.6^a^	44.55^a^	20.61^a^	269.4^a^	196.1^a^	203.8^a^	26.35^b^
HFD + L	191.2^a^	44.21^a^	20.83^a^	267.8^a^	195.6^a^	202.2^a^	26.50^b^
HFD + L + 0.1 SeNPs	136.8^b^	28.89^b^	9.730^b^	111.5^b^	106.6^b^	53.33^b^	36.81^a^
HFD + L + 0.2 SeNPs	136.9^b^	28.91^b^	9.755^b^	108.6^b^	107.0^b^	49.85^b^	37.29^a^
SEM	5.559	1.393	0.923	11.54	7.132	10.80	0.884
*P*-value	﻿< 0.001	﻿< 0.001	﻿< 0.001	﻿< 0.001	﻿< 0.001	﻿< 0.001	﻿< 0.001

*C*, control; *HFD*, high-fat diet; *L*, letrozole; *TC*, total cholesterol; *TG*, triglycerides; *LDL*, low-density lipoprotein cholesterol; *HDL*, high-density lipoprotein cholesterol; *SEM*, standard error of means

Means in the same column bearing different superscripts are significantly different at (*P* < 0.05)

### Serum Hormonal Profile

As presented in Table 3, serum levels of testosterone and LH were increased (*P* ﻿< 0.01) in the HFD + L group compared to C and HFD groups, while estradiol and progesterone concentrations were reduced (*P* ﻿< 0.01). The values of the abovementioned hormones were restored (*P* < 0.01) near to the normal levels after treatment with SeNPs at 0.1 and 0.2 mg/kg BW. No difference in the level of FSH among experimental groups.

**Table 3:** Effect of selenium nanoparticles (SeNPs) on serum testosterone, estradiol, progesterone, LH, and FSH in polycystic ovary syndrome rat model

Items	Testosterone (pg ml^−1^)	Estradiol (pg ml^−1^)	Progesterone (ng ml^−1^)	LH (IU ml^−1^)	FSH (IU ml^−1^)
C	77.08^c^	36.05^a^	7.876^a^	2.131^c^	7.039
HFD	76.78^c^	35.91^a^	7.923^a^	2.216^c^	6.911
HFD + L	242.6^a^	14.67^c^	4.564^c^	5.430^a^	6.836
HFD + L + 0.1 SeNPs	127.8^b^	28.46^b^	5.872^b^	3.487^b^	6.845
HFD + L + 0.2 SeNPs	128.0^b^	28.09^b^	5.918^b^	3.532^b^	6.905
SEM	8.247	1.090	0.186	0.171	0.040
*P*-value	﻿< 0.001	﻿< 0.001	﻿< 0.001	﻿< 0.001	0.511

*C*, control; *HFD*, high-fat diet; *L*, letrozole; *LH*, luteinizing hormone; *FSH*, follicle stimulating hormone; *SEM*, standard error of means﻿

Means in the same column bearing different superscripts are significantly different at (*P* < 0.05)

### Oxidative Stress Biomarkers and Inflammatory Cytokines

Ovarian oxidative stress was provoked in female rats upon administration of HFD and HFD + L as observed by a significant increase in LPO levels (Table 4). The ovarian concentration of MDA was elevated (*P* ﻿< 0.01) in all treated groups compared to C, while it was decreased (*P* ﻿< 0.01) in SeNP-treated groups compared to HFD + L and HFD groups. In addition, a significant decrease in GPx in the ovarian tissues was noticed and the lowest GPx levels were recorded in HFD + L and HFD groups. Treatment with both levels of SeNPs elevated the concentration of GPx compared to the control groups. Serum concentrations of inflammatory cytokines (TNF-α and IL-6) were elevated (*P* ﻿< 0.01) in PCOS-induced groups (HFD + L and HFD) compared to C group. Administration of the two levels of SeNPs mitigated the hyper-inflammation compared to untreated PCOS groups.

**Table 4:** Effect of selenium nanoparticles (SeNPs) on serum TNFα and IL-6 and ovarian concentrations of MDA and GPx in polycystic ovary syndrome rat model

Items	MDA (mmol gm^−1^)	GPx (mmol gm^−1^)	TNFα (pg ml^−1^)	IL-6 (pg ml^−1^)
C	86.02^d^	66.53^a^	13.17^d^	45.65^d^
HFD	155.8^b^	21.50^c^	53.74^b^	64.83^b^
HFD + L	176.6^a^	20.04^d^	82.40^a^	72.40^a^
HFD + L + 0.1 SeNPs	106.9^c^	49.56^b^	44.05^c^	49.76^c^
HFD + L + 0.2 SeNPs	107.6^c^	49.46^b^	43.88^c^	50.30^c^
SEM	4.661	2.454	3.074	1.465
*P*-value	﻿< 0.001	﻿< 0.001	﻿< 0.001	﻿< 0.001

*C*, control; *HFD*, high-fat diet; *L*, letrozole; *MDA*, malondialdehyde; *GPx*, glutathione peroxidase; *TNFα*, tumor necrosis factor alpha; *IL-6*, interleukin 6; *SEM*, standard error of means﻿

Means in the same column bearing different superscripts are significantly different at (P<0.05)

### mRNA Expression of Ovarian Enzymes and Proteins

**Fig. 3:**
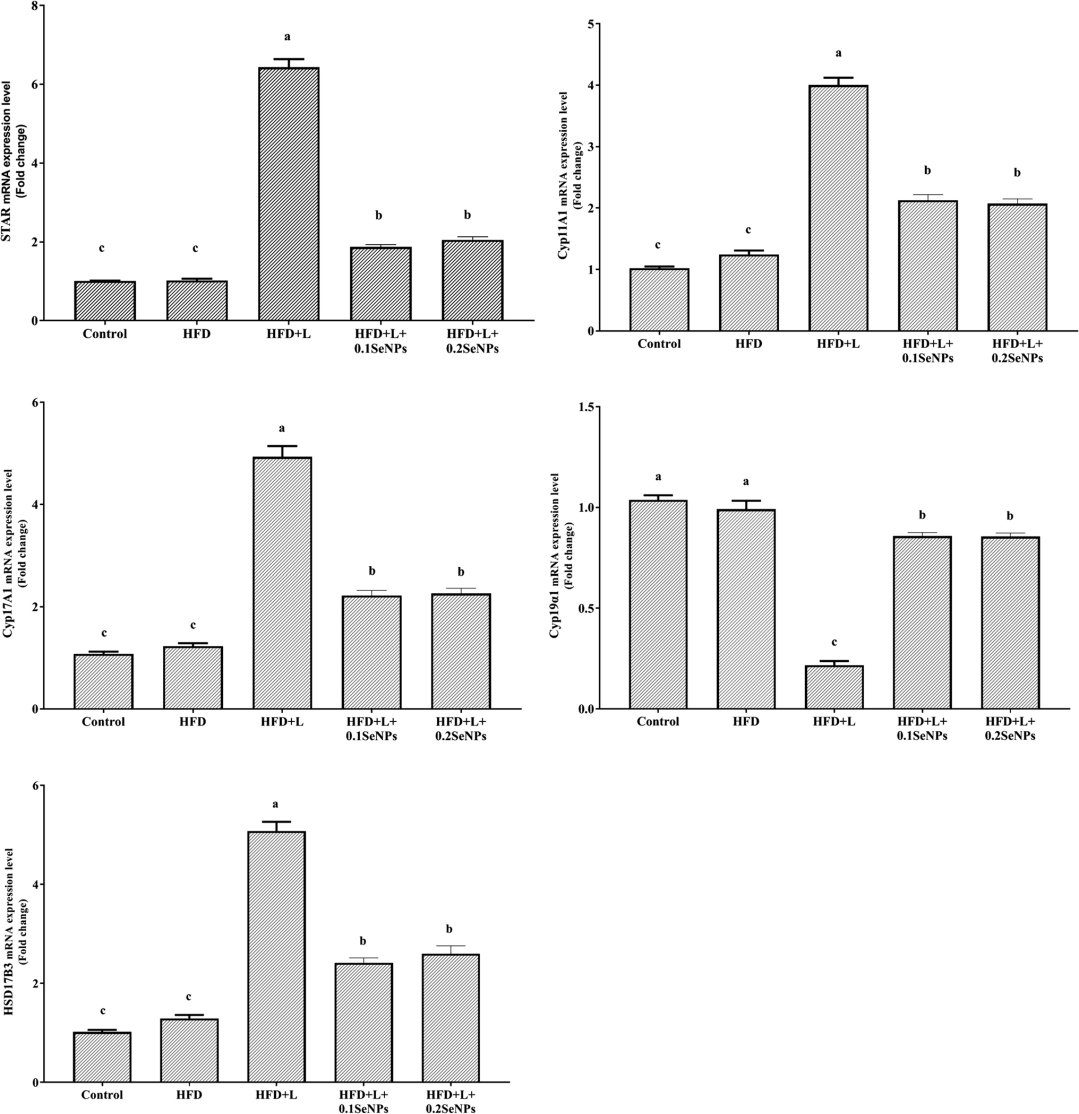
Effect of selenium nanoparticles (SeNPs) on ovarian mRNA expression of enzymes and proteins (STAR, Steroidogenic acute regulatory protein; CYP11A1, Cytochrome P450 Family 11 Subfamily A Member 1; CYP17A1, Cytochrome P450 Family 17 Subfamily A Member 1; CYP19A1, Cytochrome P450 Family 19 Subfamily A Member 1; HSD17B3, 17-beta hydroxysteroid dehydrogenase 3) involved in steroidogenesis in a polycystic ovary syndrome rat model. HFD, high-fat diet; L, letrozole. Data are presented as the mean values with their standard errors. Columns bearing different letters indicate significant changes (*P* ﻿< 0.05)

The effect of SeNPs on mRNA expression of ovarian enzymes and proteins (STAR, CYP11A1, CYP17A1, CYP19α1, and HSD17B3) PCOS rats is illustrated in Fig. 3. mRNA expressions of STAR, CYP11A1, CYP17A1, and HSD17B3 were elevated (*P* ﻿< 0.05) in PCOS rats (HFD + L group) compared to control group. However, the expression of CYP19α1 was decreased (*P* ﻿< 0.05) in HFD + L compared to C. Administration of both levels of SeNPs restored the expression of all these genes near the normal level.

### Histopathological Examination of Ovarian Tissues

**Fig. 4:**
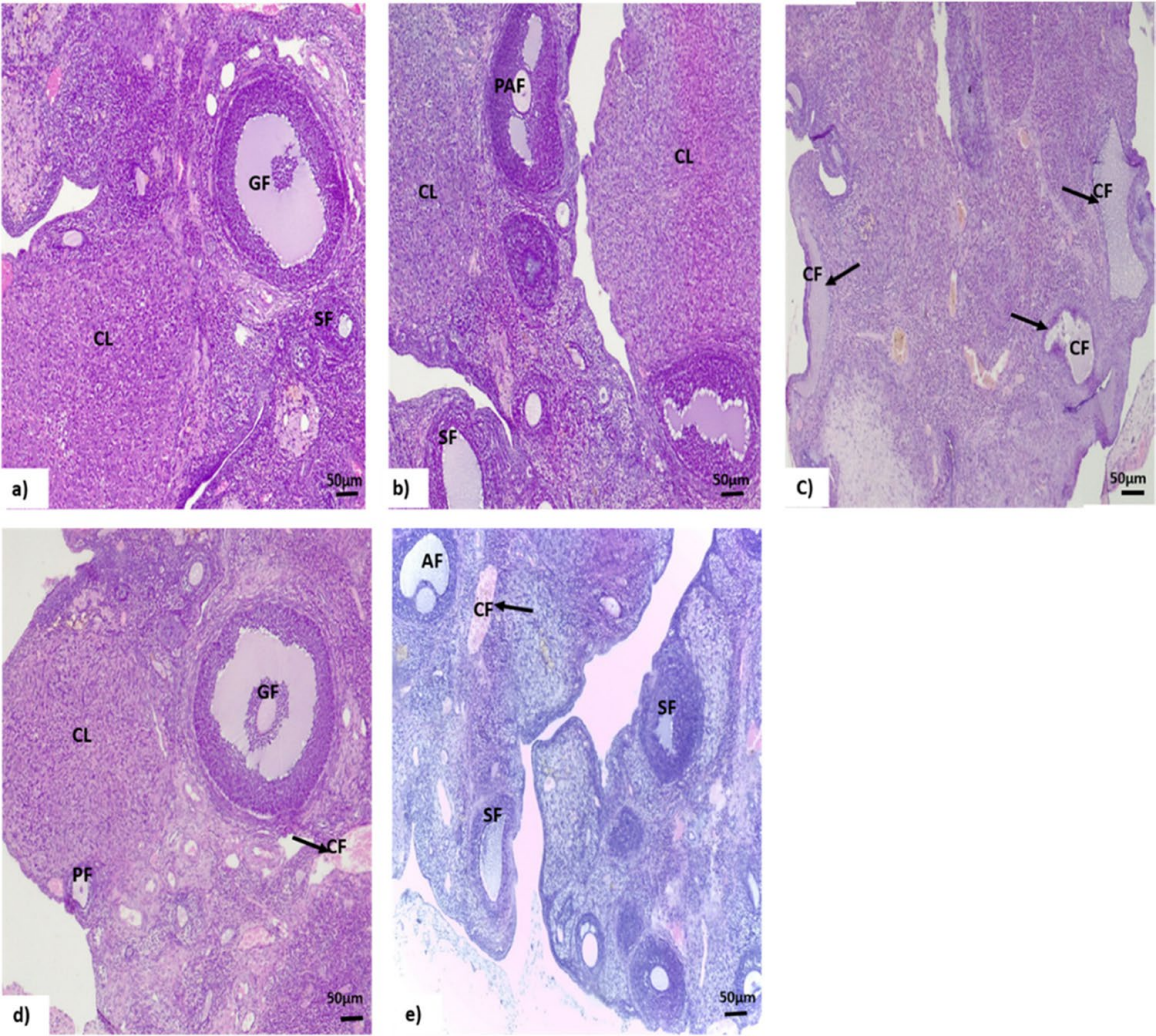
Photomicrographs of rat ovarian tissues show effects selenium nanoparticles (SeNPs) on the ovarian tissues in a polycystic ovary syndrome rat model. Experimental groups: (**a**) control, (**b**) obese (fed high-fat diet, HFD), (**c**) obese PCO (HFD + letrozole, L), (**d**) obese PCO treated with 0.1 SeNPs, (**e**) obese PCO treated with 0.2 SeNPs. (**a**, **b**) Ovarian tissue from control and obese groups shows normal architecture of ovarian tissue with follicles at different stage of development. (**c**) Ovarian tissue from obese PCO group shows the presence of multiple subcapsular cystic follicles. (**d**, **e**) Ovarian tissue from SeNP-treated groups shows normal developing follicles with decrease number of cystic follicles. GF, graffian follicle; CL, corpus luteum; SF, secondary follicle; PF, primary follicle; AF, antral follicle; PAF, pre-antral follicle; CF, cystic follicle (H&E × 100)

Histopathological examinations of ovarian tissue from all experimental groups are presented in Fig. 4. The ovarian tissues from the C and HFD groups show ovarian follicles at a different stage of development with several fresh corpora lutea and surrounded by normal ovarian stroma (Fig. 4a, b). Ovarian tissue from HFD + L group shows the presence of multiple fluid-filled subcapsular cystic follicles surrounded by a thin layer of degenerated granulosa cells and thickened theca cell layer with the absence of antral follicles or corpus luteum (Fig. 4c). Ovarian tissues from SeNP-treated groups show to some extent return of normal ovarian architecture, the appearance of different intact follicles with recently formed corpus luteum and decrease number of cystic follicles (Fig. 4d, e).

### Immunohistochemical Staining of Androgen Receptors

**Fig. 5:**
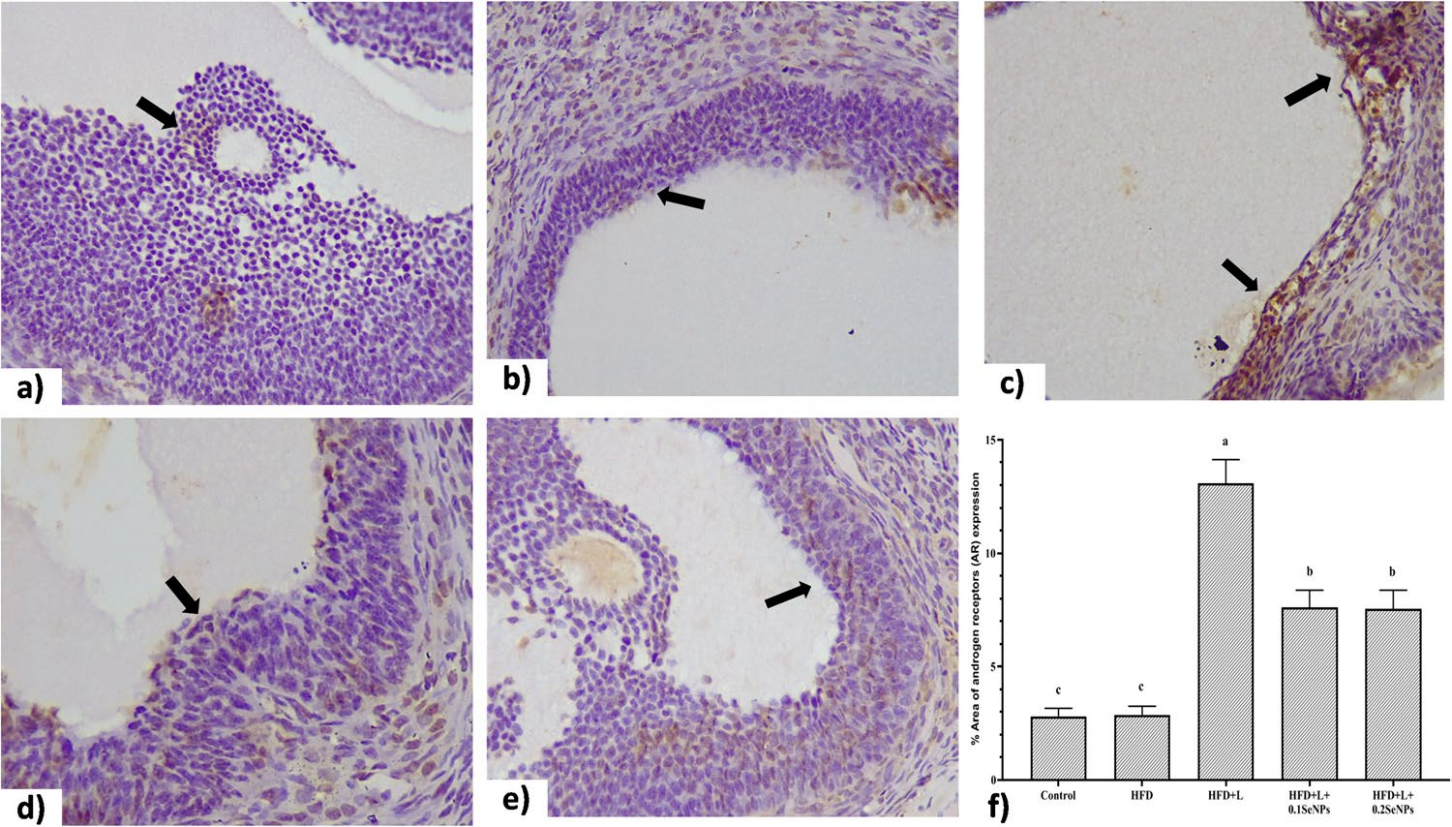
Microscopic images show effect of selenium nanoparticles (SeNPs) on the androgen receptor (AR) expression in the ovarian tissues in a polycystic ovary syndrome rat model. Immunohistochemical staining of AR in different experimental groups: (**a**) control, (**b**) obese (fed high-fat diet, HFD), (**c**) obese PCO (HFD + letrozole, L), (**d**) obese PCO treated with 0.1 SeNPs, (**e**) obese PCO treated with 0.2 SeNPs. Short arrow signifying the positive (brown) staining of the nucleus of granulosa cells indicates the expression of AR (× 400). (**f**) The statistical analysis of the area percent of AR expression among the studied groups. Data are presented as the mean values with their standard errors. Columns bearing different letters indicate significant changes (*P* ﻿< 0.05)

Immunolocalization and expression of androgen receptors in ovarian sections of POCS rats are illustrated in Fig. 5. Higher AR expression mainly in the nuclei of granulosa and theca cells in HFD + L group (Fig. 5c) when compared to C groups (Fig. 5a) revealing mild immune-expression. However, a decrease in AR expression in the corresponding ovarian cells in SeNP-treated groups (Fig. 5d, e) compared to HFD + L group. These results were further confirmed by quantitative analysis (Fig. 5f), as the % area of AR expression was increased (*P* ﻿< 0.05) in HFD + L group compared to the control, while the % area of AR expression was decreased (*P* ﻿< 0.05) in SeNP-treated groups when compared to HFD + L group.

## Discussion

In the current study, we established a young-adult rat model of HFD and L-induced PCOS showing characteristics of obesity, hyperandrogenism, lack of ovulation, and polycystic ovaries. The potential effect of intragastric administration of SeNPs on follicular development and ovulation was also assessed. To the best of our knowledge, the present study proved, for the first time, the role of SeNPs in modulating the expression of androgen receptors and action in the POCS rat model.

The estrous cycle reflects the ovarian and uterine status and hormonal physiology. Disruption in estrous cyclicity is a major distinguishing feature of PCOS rats [39]. The results of the present study revealed that PCOS rats exhibited almost identical reproductive dysfunctions and estrous cycle disruption compared to normal rats. Our results are concomitant with Dey et al. [40] and Huang et al. [15], who noticed irregular estrus cyclicity and identical reproductive dysfunctions in L or HFD + L-induced PCOS rats. In this study, the administration of SeNPs to POCs rats could normalize the oestrus cycle.

In the present study, the final BMI increased in all treated groups compared to the control. The highest value of final BMI was recorded in HFD, followed by HFD + L. The increase in body weight could also be linked to fat accumulation in the abdominal area, which might induce adipocyte dysfunction and the insulin resistance-like state in PCOS [41]. These findings agree with Huang et al. [15] and Haslan et al. [42], who reported an increase in the body weight and BMI of the HFD + L-treated rats compared to normal ones**.**

Hyperglycemia has a crucial effect on the incidence of PCOS [43]. Our results revealed that HFD and HFD + L groups exhibited elevated fasting blood glucose, insulin levels, and HOMA-IR values compared to normal group. Intragastric administration of SeNPs decreased hyperglycemia and serum insulin levels and improved insulin resistance of POCS rats, as shown by the reduction in HOMA_IR compared to the untreated PCOS ones**.** Selenium could reduce glucose levels and increase its transport due to its insulin-mimetic and insulin-like activities [44]. Furthermore, it has been reported that selenate could translocate glucose transporters, such as GLUT-1 and GLUT-2, to the surface of many membranes and enhance its transportation and uptake in adipocytes of rats [45]. Also, Se could lower the glucose level via acceleration of its excretion through the kidney in rats, when added at 0.1 mg/kg in the nanoform [46], and activating Akt and other kinases, such as p70S6 kinase, which are responsible for insulin signaling [47]. In line with our results, El-Borady et al. [48] and Vural et al. [49] demonstrated that STZ-induced diabetic rats exhibited reduced blood glucose levels in response to SeNPs (at 0.5 mg/ml) and sodium selenite (at 0.4 mg/kg) when compared to untreated diabetic ones.

PCOS has been linked to dyslipidemia. The current study showed that TC, TG, and LDL levels increased while HDL decreased in untreated POCS rats. Similar metabolic dysregulation was observed by Ibrahim et al. [50], who reported that the increase in circulating levels of triglycerides might be attributed to elevated androgen concentrations induced by letrozole. This effect might be attributed to disturbances of both enzymes and proteins involved in lipid metabolism due to hyperandrogenemia [51]. Lipid profile parameters were normalized after treatment with SeNPs. Similar findings have been reported earlier by Butt et al. [24] when SeNPs administrated at 50 and 100 mg/kg. The hypolipidemic effect of SeNPs may be attributed to the enhanced catabolism of cholesterol to bile acids and the suppression of cholesterol synthesis and LDL receptor activity [19]. In addition, Zhang et al. [52] stated that supplementation of 0.05 and 0.1 mg Se/kg bw increased gene expression levels of cholesterol esterification enzyme and decreased the expression of endogenous cholesterol synthesis enzyme (HMGR).

Steroid hormones in the follicular fluid play an important role in the physiology of follicular growth, oocyte maturation, and ovulation [53]. Recent studies demonstrated that one of the main findings of PCOS women and PCOS animal models is the disturbance in sex steroid hormones [54]. Our results revealed that the serum levels of testosterone and LH hormones were increased while estradiol and progesterone levels were decreased in HFD + L when compared to the control. No significant change was observed in the serum level of FSH. These results are similar to those reported by Butt et al. [24] and Ryu et al. [54].

The hyperandrogenemia in untreated PCOS rats was attributed to blocking aromatization of testosterone to estradiol, ensuing in elevated androgen levels and may lead to elevated LH:FSH ratio [55]. In addition, increased serum cytokines and hyperglycemia concomitant with using letrozole may also elevate testosterone and LH levels. According to Matsuzaki et al. [56], the abnormal high level of LH stimulates the ovarian theca interna cells to produce excessive androgens and induces apoptosis in the follicular granulosa cells, leading to the polycystic ovary. Furthermore, hyperglycemia or glucose intolerance may indirectly suppress the synthesis of hepatic sex hormone-binding globulin synthesis (SHBG), leading to increased serum levels of unbound steroid hormones which in turn modulate the activity of the key regulatory enzyme of androgen biosynthesis, P450c17, in the ovarian theca interna and interstitial cells resulting in elevated androgen levels in PCOS women. SeNPs treatment in the present study reversed the increment in the testosterone and LH levels and improved estradiol and progesterone levels. This could be explained by the ability of SeNPs to ameliorate the altered cystic follicles, antral follicles, and corpus luteum in PCOS rats [24]. Moreover, it has been reported that high levels of testosterone contribute to the pathogenesis of PCOS and repression of its excessive production by SeNPs administration may have beneficial effects on PCOS disturbances [28].

PCOS is also associated with metabolic disturbances, chronic inflammation, and oxidative stress, which alter ovarian steroidogenesis, promoting androgen production and stimulating polycystic ovaries. In the present study, the untreated PCOS rats demonstrated higher ovarian oxidative stress marker (MDA) and serum cytokines (TNF-α and IL-6) and a decline in ovarian antioxidant defense marker (GPx) when compared to normal control. Virshette et al. [57] reported that lipid peroxidation stimulates the oxidative free radical damage of fatty acid cell membranes ensuing cell necrosis and inflammation. Furthermore, Paszkowski et al. [58] reported that the follicular fluid of tobacco-smoking women has a marked decrease in the activity of GPx while the fertilized oocytes were found to have higher GPx activity than non-fertilized ones as in untreated POCS rats. Treatment with SeNPs exerts a beneficial potential in obese PCOS rats via decreasing the ovarian level of MDA and serum inflammatory cytokines and increasing the ovarian level of GPx compared to HFD + L group. Previous studies confirmed that SeNPs have potent antioxidant and anti-inflammatory characteristics and reported that treatment with SeNPs (at 0.5 mg/ml) and sodium selenite (at 0.4 mg/kg) markedly decreased diabetes-induced lipid peroxidation in rats [48, 49]. SeNPs can scavenge ROS, attributed to their incorporation in one of the most important antioxidant enzymes, GPx. Dkhil et al. [59] reported that GPx activity indicates the level of Se and selenoprotein in a body.

The main treatment technique for female infertility depends on improving female reproductive function by promoting ovarian steroidogenesis. In the present study, the mRNA expression of STAR, CYP11A1, CYP17A1, and HSD3B1 was increased, whereas the mRNA expression of CYP19α1 was reduced in untreated PCOS animals. The change in steroidogenic markers could be correlated with the alteration in hormones. According to Diamanti-Kandarakis et al. [60], high levels of insulin stimulate thecal androgen production and directly affect ovarian steroidogenesis. The high levels of LH and insulin synergistically affect STAR expression, increasing its level by co-binding to the STAR promoter region [61]. This leads to a shift in STAR expression profile, resulting in altered enzyme activity and disturbed steroidogenesis. Moreover, Kafali et al. [28] have reported dysfunctional aromatase activity in PCOS women**.** Aromatase is encoded by the Cyp19α1 gene and plays a key role in the normal progression of the menstrual/estrous cycle in rats with PCOS [62]. The activity of aromatase in the granulosa cells in polycystic ovaries is low, ensuing in an imbalance in the production of estrogen and androgen [63]. These findings are consistent with previous data demonstrated by Munawar Lone et al. [64]. However, treatment of PCOS rats with SeNPs recovered the reduction in aromatase expression. Several studies revealed the potential of letrozole to provoke PCOS in a rat model [50]. Letrozole inhibits aromatase enzyme activity, resulting in excessive accumulation of androgens in the ovaries due to the minimal conversion of androgens to estrogens [65]. The reduction in aromatase expression in PCOS ovaries could be linked to promoter hypermethylation which may be involved in the pathogenesis of PCOS [66]. In addition, testosterone was reported to have a key role in the downregulation of aromatase in PCOS with the downregulation of mRNA and protein levels of aromatase in cultured luteinized granulosa cells [67]. Chien et al. [68] reported that pregnenolone was synthesized in the inner mitochondrial membrane from cholesterol by CYP11A1. CYP17A1 converts pregnenolone into dehydroepiandrosterone and the further formation of androstenedione [69]. Finally, TES is formed from androstenedione, and HSD17B3 catalyzes the respective back-reaction [70]. Aromatase (CYP19A1) catalyzes TES’s irreversible conversion into E2 [71].

The histopathological examination of the obese PCOS ovaries showed the presence of multiple fluid-filled subcapsular cystic follicles surrounded by a thin layer of degenerated granulosa cells with the absence of antral follicles or corpus luteum. Similar findings were reported by Ibrahim et al. [50], who demonstrated that PCOS ovaries showed multiple ovarian cysts with a lack of corpus luteum, growing follicles, oocytes, granulosa, and theca cell layers. The decline in the number of corpus lutea may be attributed to the anovulation of PCOS. Also, in accordance with current results, recent reports showed that PCOS ovaries exhibited numerous ovarian cysts and small follicles in the early developmental stage and the absence of corpora lutea. The follicular walls of the cystic follicle contained a very thin layer of flattening granulosa cells [72]. Kafali et al. [28] reported that the anovulatory state might be due to active FSH and LH levels and the reduced interplay between cells of ovaries, such as theca cells and granulosa cells. Baravalle et al. [73] reported a thin layer of granulosa cells, resulting in hyperplasia of theca cells. In contrast, the treatment of PCOS rats with SeNPs returned the ovarian tissue to some extent to normal architectures with the appearance of follicles at different stages of development and a decreased number of cystic follicles. Several reports revealed similar results [24].

The effects of ovarian steroid hormones are mediated through their interaction with specific receptors and steroidogenic enzymes and perform several important actions related to ovarian functions [74]. The results of the current study showed higher AR protein immunoexpression within the nucleus of granulosa cells in obese PCO groups compared to the control groups, which revealed mild immune expression. These results are concomitant with the findings of Dey et al. [40], who showed that AR expression was significantly increased in letrozole-treated rats. Manneras et al. [75] also observed an upregulation of the AR due to the accumulation of endogenous testosterone resulting in alteration in the ovary. Consistent with these findings, when prepuberal rats were administered testosterone propionate at 5 days of age, the ovarian nuclear AR expression was also increased [76]. On the contrary, ovaries of SeNPs (50 and 100 mg/kg) treated obese PCOS rats exhibited decreased AR protein expression suggesting the anti-androgenic effect of SeNPs [24].

## Conclusion

Results of the present study demonstrated that SeNPs had a beneficial potential in a rat model of HFD-L-induced PCOS. The treatment with SeNPs restored the estrus cyclicity, decreased hyperglycemia and insulin resistance, improved lipid profile, reduced the elevated levels of serum testosterone, and LH and recovered the ovarian cysts. Furthermore, SeNPs normalized the mRNA expression of genes involved in steroidogenesis and decreased the immunoexpression of AR. Therefore, it can block the vicious circle initiated by hyperandrogenemia and improve the metabolic, endocrine, and reproductive dysfunctions associated with PCOS cases.

## Data Availability

The datasets generated and analyzed during the current study are presented in the manuscript.
